# Identifying Barriers and Pathways to Care Among College Students at Risk of or Diagnosed with First Episode Psychosis

**DOI:** 10.3390/psychiatryint6010015

**Published:** 2025-02-12

**Authors:** Annette S. Crisanti, Justine L. Saavedra, Sam Barans, Perla M. Romero, Natasha Dark, Bess Friedman, David T. Lardier, Juan Bustillo, Mauricio Tohen, Rhoshel Lenroot, Cristina Murray-Krezan

**Affiliations:** 1 Department of Psychiatry and Behavioral Sciences, Health Sciences Center, University of New Mexico, Albuquerque, NM 87131, USA; 2 School of Medicine, Health Sciences Center, University of New Mexico, Albuquerque, NM 87131, USA; 3 Department of Medicine, School of Medicine, University of Pittsburgh, Pittsburgh, PA 15261, USA

**Keywords:** psychosis, college students, coordinated specialty care, barriers to care, pathways to care

## Abstract

Prolonged untreated psychosis worsens outcomes, thus motivating the study of pathways and perceived barriers to care, especially for high-risk age groups like college students. The primary objective of this study was to explore pathways to coordinated specialty care (CSC) and perceived barriers to care in college students at high risk for psychosis or with first-episode psychosis and determine any association between them. Twenty-four college students enrolled in CSC completed the Circumstances of Onset and Relapse Schedule and Barriers to Seeking Psychological Help Scale (BSPHS). Non-parametric tests were used for two-group analyses, and medians and interquartile ranges (IQR) were calculated. The median number of total contacts along the pathway to CSC was 5.0 (IQR = 5.2), with more psychiatric contacts (Mdn. = 3.0, IQR = 2.2) than non-psychiatric contacts (Mdn. = 2.0, IQR = 3.0). Students whose first contact was with non-psychiatric services had longer pathways to care overall, with a higher median number of total psychiatric as well as non-psychiatric contacts relative to students whose first contact was with psychiatric services. With the highest possible total BSPHS score being 5, the median score was 2.7 (IQR = 0.8). Targeted psychosis literacy training for non-mental health professionals and anti-stigma campaigns for college students may help reduce the duration of untreated psychosis.

## 1. Introduction

There is a strong association between the duration of untreated psychosis (DUP) and poor outcomes, including the extent of remission and response to coordinated specialty care (CSC) [1–3]. Towards the goal of reducing DUP, research has focused on pathways to CSC and factors that contribute to delays along the pathways, such as barriers to care among individuals at clinical high risk of psychosis (CHRp) or experiencing a first episode of psychosis (FEP) [4,5]. Because the first symptoms of psychosis most often surface among college-age young adults, compared to any other age group [6,7], exploring pathways and barriers to CSC among this population are important areas for research to identify interventions that minimize treatment delay.

### 1.1. Pathways to Care

There has been extensive research on pathways to care for individuals with FEP and, to a lesser extent, among those at CHRp [8–12]. Pathways to care research typically examines initial self-perceived symptoms through referral to CSC as well as the “sequence of contacts with individuals and organizations prompted by the distressed person’s efforts, and those of his or her significant others, to seek help as well as the help that is supplied in response” [13]. Cabassa et al. [10] found that the median number of professional contacts from the onset of FEP to entry into CSC was five. A systematic review on pathways to care among those at CHRp found that the number of contacts along the pathway to care ranged between 0 and 9, with a pooled mean of 3.22 [8]. With respect to the type of first contact, primary care physicians tend to be the most likely type of first contact for the largest proportion of patients [4,9,12].

While racial/ethnic disparities have been identified in the research on factors that influence pathways to care, the studies have mostly focused on Black compared to White patients, and Hispanics have been underrepresented in the study populations [14–17]. In a study of characteristics of Hispanics referred to CSC, Friedman et al. [18] found that Hispanics were more likely than non-Hispanics to be referred from either inpatient or outpatient mental health providers and not from other community resources. With respect to sex differences and pathways to care, the research has also been limited and findings inconsistent [19]. For example, Bhui et al. [4] found that the first contact among males enrolled in CSC was most likely to be the criminal justice system, which contributed to longer DUP. In contrast, while research from Large et al. [20] supports the finding that male mean DUP is generally longer, they also mention that in lower-income countries, females experience longer mean DUP than males.

### 1.2. Barriers to Care

While there has been extensive research on perceived barriers to mental health care among college-age young adults [21], very few studies have focused on perceived barriers to CSC. Of those few studies that have been conducted, the research has been mostly qualitative and has focused on barriers experienced by family members rather than those diagnosed with early psychosis [5,22]. With psychosis being associated with hallucinations, delusions, and disordered thinking and behaviors, young adults at CHRp or with FEP may report greater or different barriers to care compared to young adults with other mental illnesses such as depression or anxiety. Using data collected through the Healthy Minds Study, a survey of 62,025 students across 79 different universities in 2018–2019, Borgogna et al. [23] found that students who self-reported being treated for psychosis described more barriers to treatment compared to students who self-reported being treated for depression. For example, compared to their counterparts, students who self-reported being treated for psychosis were significantly more likely to report not knowing where to go for treatment (17.6% vs. 8.2%).

### 1.3. Study Objectives

The primary objective of this study was to explore pathways to care and perceived barriers to care among a cohort of college students identified as CHRp or FEP enrolled in a CSC treatment program and to determine whether there was an association between the two. Our secondary objective was to explore whether pathways to care and perceived barriers to care varied by sex at birth or ethnicity. The stratified analysis was influenced by the limited and inconsistent findings related to these two variables and the heterogeneity of our study population. Nearly half of the participants in our study identified as Hispanic, which is representative of the state in which this study was conducted.

## 2. Materials and Methods

### 2.1. Study Design

Upon admission to the UNM CSC program, participants were invited to take part in a study to determine pathways to CSC and perceived barriers to care. After enrollment in the CSC program, participants consented to participate in the study and to use the data generated for research.

### 2.2. Study Sample

The sample (*N* = 24) included participants who were enrolled in the University of New Mexico (UNM) Department of Psychiatry and Behavioral Health Sciences CSC program between August 2020 and July 2023. Most of the participant recruitment took place during the first two years. The CSC program included separate clinics for patients identified as CHRp or FEP. Seventeen (71%) participants were enrolled in the program that served CHRp, and seven (29%) participants were enrolled in the program that served FEP. With respect to the primary clinical diagnosis determined by a psychiatrist, 14 (58%) participants were diagnosed with a non-affective psychotic disorder, and 10 (42%) were diagnosed with an affective disorder with psychotic features.

### 2.3. Procedures and Measures

Following consent, data were collected on (1) basic demographics, (2) pathways to CSC, and (3) perceived barriers to care. Demographics included age, race, ethnicity (Hispanic or non-Hispanic), sex at birth (male or female), and level of education. All data were collected via face-to-face interviews or HIPAA-compliant Zoom.

Barriers to care data were collected using the Barriers to Seeking Psychological Help Scale (BSPHS), developed to measure barriers related to psychological help-seeking for college students [24]. While this scale has yet to be used with a population experiencing psychosis, it has been used with students experiencing anxiety and depression [24]. At the time of conducting this study, a barrier to care measure specific for FEP or CHRp populations did not exist. The BSPHS has established validity and reliability, and the Cronbach Alpha internal consistency coefficients obtained from the different samples were mostly above 0.7 [24]. The scale includes 17 items measured on a 5-point Likert-type scale ranging from strongly disagree to strongly agree. The scale has five subscales, including (1) fear of being stigmatized by society, (2) trust in mental health professionals, (3) difficulties in self-disclosure, (4) perceived devaluation, and (5) lack of knowledge.

The Circumstances of Onset and Relapse Schedule (CORS) was used to measure pathways to CSC [25]. The CORS was developed specifically for people experiencing psychosis and has been used for this purpose in multiple studies [26]. The tool traces the process of seeking treatment by identifying step-by-step contacts with medical, mental health, and/or community services in the pathway toward enrollment in CSC. Participants were asked if they had ever sought a particular service or professional for mental health problems, and if so, the approximate date of that encounter. The services/professionals asked about included family doctor, walk-in clinic, emergency room, hospital admission, counselor/psychotherapist, psychologist, psychiatrist, school counselor, clergy, or other homeopathic remedy. The reasons for seeking out help and the treatment provided (medication, counseling, referral, evaluation/consult, admission, unknown) were also recorded. The CORS has well-established psychometric properties and has been frequently used to measure pathways to care among populations with FEP [25,27].

### 2.4. Analyses

BSPHS total and subscale scores were determined by taking the mean of responses to all items for the entire scale and the mean of item responses for each subscale, respectively. Higher scores indicate higher perceived barriers, with the following cutoffs identified by those who created the BSPHS: very low: 1.00–1.79, low: 1.80–2.39, moderate: 2.40–3.19, high: 3.20–4.19, and very high: 4.20 (personal communication with Nursel Topkaya, dated 26 October 2022).

The pathways to care data from the CORS were synthesized using a visual route timeline (VSR). The VSR was used to summarize the date, number, and sequence of help-seeking contacts. By analyzing the VSR, we created categories for first contact on the pathway to care prior to enrollment in CSC based on dates of contact. The total number of contacts along the pathway was calculated by tallying the total number of times the participant sought help for mental health problems before being connected to CSC services. Contacts were classified into psychiatric vs. non-psychiatric based on the type of services/professional sought. Contacts with a psychiatrist, psychologist, or mental health counselor and psychiatric hospitalization were classified as psychiatric contacts, while all other categories (i.e., seeking help from a clergy member or school counselor, going to a family doctor, walk-in clinic, or the emergency room for mental health problems, and any other contact) were classified as non-psychiatric. Participants were sorted into groups for comparison based on first contact (psychiatric vs. non-psychiatric). Participants were also sorted into groups for comparison based on whether they had more psychiatric vs. non-psychiatric contacts. Five participants with an equal number of psychiatric and non-psychiatric contacts were each randomly assigned to one of the two groups for the sake of analyses requiring dichotomous variables. The median time between the date at which participants first noticed that something was “wrong” and the time of enrollment into CSC was also calculated for all categorical groups and labeled as the duration of untreated distressing symptoms (which is different than the duration of untreated psychosis).

We report medians and quartiles for barriers and pathway variables for the entire sample and also stratified across ethnicity (Hispanic vs. Non-Hispanic), sex at birth (female vs. male), more contacts (psychiatric vs. non-psychiatric), and first contact (psychiatric vs. non-psychiatric). Quantitative analyses were performed in R version 4.3.0 (2023) [28].

## 3. Results

A total of 26 participants enrolled in the study, and 24 completed the BSPHS and CORS. The median age was 21.0 (IQR = 3.0); the majority were female (*n* = 16, 67%), white (*n* = 19, 79%), and nearly half identified as Hispanic (*n* = 10, 42%). In addition, 42% (*n* = 10) of participants were in their 3rd or 4th year of undergraduate studies (see Table 1).

Pathways to Care: Overall, the median number of total contacts was 5.0 (IQR = 5.2). The median number of psychiatric contacts was 3.0 (IQR = 2.2), and the median number of non-psychiatric contacts was 2.0 (IQR = 3.0). The median duration of untreated distressing symptoms was 1.2 years (IQR = 3.0) (see Table 2).

Participants were evenly split in terms of their type of first contact, with the initial contact for 50% (*n* = 12) being psychiatric and for the other 50% (*n*= 12) being non-psychiatric. Among those whose first contact fell within the psychiatric grouping, the most common first contact was a counselor or psychotherapist (*n* = 8, 33%). Over the duration of their pathway to care, 58% (*n* = 14) had predominantly psychiatric contacts, while 42% (*n* = 10) had more non-psychiatric contacts.

Participants whose first contact was psychiatric had fewer contacts overall before being connected with CSC. The median number of total contacts for participants whose first contact was psychiatric was 4.0 (IQR = 3.0) compared to 7.0 (IQR = 4.3) for participants whose first contact was non-psychiatric. Participants whose first contact was psychiatric experienced a median of 1.5 (IQR = 2.0) non-psychiatric contacts in their pathway to care, which was fewer than the 3.5 (IQR = 2.0) median non-psychiatric contacts experienced by those whose first contact was non-psychiatric.

The median of 3.0 non-psychiatric contacts (IQR = 2.0) experienced by females was higher than the median of 1.0 (IQR = 0.5) experienced by males. Total number of contacts, type of first contact (psychiatric vs. non-psychiatric), and number of contacts (psychiatric vs. non-psychiatric) did not vary between Hispanics and non-Hispanics.

Perceived Barriers to Care: The median total BSPHS score was 2.7 (IQR = 0.8). The subscale “Trust in Mental Health Clinicians” was the highest median subscale score (Mdn. = 2.9, IQR = 1.3), followed by “Fear of Stigma” (Mdn. = 2.8, IQR = 1.3), “Self-Disclosure Difficulty” (Mdn. = 2.5, IQR = 0.9), “Lack of Knowledge: (Mdn. = 2.3, IQR = 1.0), and “Perceived Devaluation” (Mdn. = 2.0, IQR = 1.2). No differences were observed between Hispanics and non-Hispanics or between males and females on the total BSPHS score or any of the subscales.

Relationship Between Pathways to Care and Perceived Barriers to Care: Participants whose first contact was non-psychiatric scored slightly higher on the BSPHS Trust in Mental Health Clinicians and Perceived Devaluation subscales, reflecting less trust in mental health clinicians and more negative thoughts about themselves if they need to or when they receive psychological help.

## 4. Discussion

Participants experienced a median of 5.0 contacts (IQR = 5.2) along their pathway to CSC. The median number of psychiatric contacts was 3.0 (IQR = 2.2), with the median number of non-psychiatric contacts not far behind at 2.0 (IQR = 3.0). This finding is consistent with other studies on pathways to CSC [10]. Participants were more likely (58%) to have contact with a psychiatrist, psychologist, counselor, or psychiatric hospitalization (classified as a psychiatric contact) along their pathway to CSC compared to non-psychiatric contacts (42%). Noteworthy was those participants whose first contact was non-psychiatric faced longer pathways to care (in terms of the number of overall contacts)—with some making as many as 15 contacts with the health care system prior to being connected with CSC. This is consistent with other research that has found a relationship between first contact with a primary care provider and longer referral delays to specialized services [29]. This finding suggests the need for psychosis literacy training, particularly among family doctors, emergency room providers, clergy members, and school counselors. A 2017 study that examined psychosis literacy training provided to professionals who work with young people found that the training increased “(1) knowledge of psychosis; (2) ability to recognize signs and symptoms of psychosis; (3) awareness of how to access services; and (4) confidence in providing help to people experiencing psychosis” [30].

General practitioner training programs on early psychosis have been demonstrated to be effective in increasing referrals [31] and should be extended to other individuals in the community that young adults have easier access to and may feel more comfortable reaching out to when in distress. A 2021 study found that a cohort of 81 Latinx community members who attended facilitated workshops by two community health educators demonstrated increased psychosis literacy in several domains and competency post-course in recognizing the symptoms of psychosis in a case vignette and appropriately seeking out services [32]. This shows that training more community members on how to recognize psychosis, especially in Latinx communities that were more likely to have a non-psychiatric first contact in this study, can help reduce DUP.

In a recent study on factors associated with referral from acute hospital-based settings and initial engagement in CSC, females were less likely to be referred to CSC compared to males [14]. While the relationship between this finding and our finding that females were more likely than males to have non-psychiatric contacts along their pathway to CSC is unclear (Mdn. = 3.0 vs. 1.0, respectively), one speculation is that females may end up reaching out to non-psychiatric contacts to receive help if their mental health concerns were, at some point, dismissed by a mental health provider. Psychosis literacy training among non-psychiatric contacts may also have the potential to decrease the gender-based disparity that has been reported in terms of women being less likely than men to access and receive specialized care [33].

The total median BSPHS score and all of its subscale scores were higher for participants whose first contact was non-psychiatric compared to participants whose first contact was psychiatric. Once again, this finding suggests that psychosis literacy training and public health campaigns that reduce stigma for undergraduate students is warranted, especially given the previously mentioned association between the first contact being non-psychiatric and greater number of contacts overall. While there is little to no research on the effectiveness of psychosis literacy training among undergraduate students, general mental health literacy training among undergraduate students has been shown to increase recognition of mental illness, help-seeking efficacy, and help-seeking attitudes [34]. Noteworthy was that the subscale “Trust in Mental Health Clinicians” had the highest median subscale score (2.9, IQR = 1.3) among all the subscales. While the reported mistrust of mental health clinicians may be a result of symptoms typical of psychosis (e.g., anosognosia and/or paranoia delusions), it is worth further exploration.

Almost half of the enrolled participants were Hispanic, accurately representing the diversity of college students in New Mexico. Our data indicate that Hispanics may be more likely to have a first non-psychiatric contact than non-Hispanic participants. Hispanic participants more commonly initiating engagement with non-psychiatric health care is consistent with a recent study published by Oluwoye and Weeks [15], who reported that “ethnoracial minoritized family members disproportionately made early contact with informal resources (e.g., religious/spiritual leaders, friends, online support groups) on the pathway to care compared to non-Hispanic white family members who tended to contact formal resources”.

A limitation of our study was the small sample, undermining the statistical power of our study where we chose not to assess the existence of significant associations or differences. While we were able to measure the time between the date at which participants first noticed that something was “wrong” and the time of enrollment into CSC, this is not a measure of DUP. As a result, we were unable to look at the relationship between DUP and barriers or pathways to care. Finally, the last challenge with our research relates to our measure of barriers to care. While the BSPHS was developed for college students, it has not been tested in populations with early psychosis. People experiencing psychosis may experience different barriers to care than what was listed on the BSPHS. While we provided an opportunity to our study participants to identify additional barriers to care not listed on the BSPHS, no additional barriers to care were reported. The development of a barriers to care scale specific to individuals with early psychosis is warranted. While we did not observe this, individuals with FEP or CHRp may even experience difficulty in reporting barriers to care in that their delusions might make them distrustful of research staff. Because the BSPHS does not include all possible barriers, our understanding of the perceived barriers to care, specifically those experienced by students with psychosis, may be improved by incorporating a qualitative component into our future research [35]. Finally, the differences between groups identified in this study and the interesting trends are hypothesis-generating for future larger studies adequately powered to detect differences in pathways and perceived barriers to care among members of different demographic groups.

## 5. Conclusions

Our findings highlight the potential benefits that could be achieved by having targeted strategies aimed at improving the psychosis literacy of non-psychiatric providers, community members, and students, as well as outreach to address stigma among these populations. Training university staff members who have contact with a large number of students, such as resident advisors or professors, could help identify students experiencing psychosis who are not actively seeking treatment. Future research should explore the extent to which other variables impact perceived barriers and pathways to care, including, for example, economic status, access to health care insurance, and growing up in a rural or urban community (which vary in terms of type and access to services) among college students with CHRp or FEP. Studies that collect qualitative data, in combination with quantitative data, on pathways and barriers to care could help shed light on the unique experience of college students experiencing psychosis.

## Figures and Tables

**Table 1.** Demographics and clinical characteristics (*N* = 24).

Demographics	
	Median (IQR)
Age in years	21.0 (3.0)
	Number (%)
Sex	
Female	16 (67)
Male	8 (33)
Race *	
White	19 (79)
Other	5 (21)
Ethnicity	
Non-Hispanic	13 (54)
Hispanic	10 (42)
Level of Education **	
First/Second-Year Undergraduate	7 (29)
Third/Fourth-Year Undergraduate	10 (42)
Unknown/Other	7 (29)

* Note: other races consisted of Black, Asian, Hawaiian or Pacific Islander, and more than one race.

** Note: other levels of education consisted of graduate students.

**Table 2.** Median scores (and quartiles) on barriers and pathways to care variables stratified by groups.

	BSPHS	BSPHS Fear of Stigma	BSPHS Trust in Mental Health Clinicians	BSPHS Self Disclosure Difficulty	BSPHS Perceived Devaluation	BSPHS Lack of Knowledge	Number of Contacts	Number of Psychiatric Contacts	Number of Non-Psychiatric Contacts	Duration of Untreated Distressing Symptoms (Years)
Entire Sample (*N* = 24)	2.7 [2.2, 3.0]	2.8 [2.2, 3.5]	2.9 [2.0, 3.3]	2.5 [2.0, 3.0]	2.0 [1.9, 3.1]	2.3 [2.0, 3.0]	5.0 [2.8, 8.0]	3.0 [1.8, 4.0]	2.0 [1.0, 4.0]	1.2 [0.3, 3.3]
Ethnicity										
Hispanic (*n* = 13)	2.6 [2.0, 3.0]	2.4 [1.9, 3.3]	2.6 [2.0, 3.3]	2.8 [2.1, 3.3]	2.0 [1.8, 3.3]	2.0 [2.0, 2.3]	5.0 [2.5, 8.0]	3.0 [1.3, 3.8]	2.5 [2.0, 4.0]	1.0 [0.3, 2.6]
Non-Hispanic (*n* = 10)	2.8 [2.6, 3.0]	3.0 [2.5, 3.5]	3.0 [2.5, 3.3]	2.3 [2.3, 3.0]	2.3 [2.0, 3.0]	2.3 [2.3, 3.0]	5.0 [3.0, 7.0]	3.0 [2.0, 4.0]	2.0 [1.0, 4.0]	1.2 [0.3, 2.6]
Sex										
Female (*n* = 16)	2.7 [2.2, 3.0]	2.9 [2.3, 3.5]	2.4 [1.9, 3.3]	2.7 [2.3, 3.1]	2.0 [1.9, 2.8]	2.2 [2.0, 3.1]	6.0 [2.8, 8.8]	3.0 [1.0, 4.3]	3.0 [2.0, 4.0]	0.9 [0.3, 3.3]
Male (*n* = 8)	2.7 [2.2, 2.9]	2.6 [1.9, 3.4]	3.0 [2.7, 3.3]	2.2 [1.5, 3.0]	2.5 [1.9, 3.1]	2.3 [2.3, 2.7]	4.5 [2.8, 5.0]	2.5 [2.0, 3.3]	1.0 [0.8, 1.3]	1.3 [0.3, 3.1]
More Contacts										
More Psychiatric Contacts (*n* = 14)	2.7 [2.1, 2.8]	2.8 [1.8, 3.4]	2.8 [2.1, 3.2]	2.7 [2.3, 3.0]	2.0 [1.8, 2.9]	2.3 [2.0, 2.6]	5.0 [3.3, 7.8]	3.5 [2.3, 5.0]	1.5 [0.3, 3.0]	1.2 [0.3, 3.5]
More Non-Psychiatric Contacts (*n* = 10)	2.9 [2.4, 3.0]	2.8 [2.3, 3.4]	3.3 [2.0, 3.3]	2.3 [2.0, 3.2]	2.3 [2.0, 3.4]	3.0 [2.1, 3.6]	5.0 [2.3, 7.8]	1.5 [1.0, 2.8]	3.0 [2.0, 4.8]	0.7 [0.2, 3.0]
First Contact										
First Contact: Psychiatric (*n* = 12)	2.4 [1.9, 2.8]	2.8 [1.5, 3.1]	2.3 [1.9, 3.1]	2.5 [1.8, 3.0]	2.0 [1.7, 2.8]	2.3 [2.0, 2.8]	4.0 [2.0, 5.0]	2.5 [2.0, 4.0]	1.5 [0.0, 2.0]	1.2 [0.3, 3.3]
First Contact: Non-Psychiatric (*n* = 12)	2.8 [2.6, 3.0]	2.8 [2.5, 3.5]	3.1 [2.6, 3.3]	2.5 [2.3, 3.3]	2.2 [2.0, 3.7]	2.3 [2.0, 3.4]	7.0 [4.5, 8.8]	3.5 [1.0, 4.3]	3.5 [2.0, 4.3]	0.8 [0.3, 3.1]

## Data Availability

The data presented in this study are available on request from the corresponding author due to privacy and IRB restrictions.

## Abbreviations

The following abbreviations are used in this manuscript:

CSC: coordinated specialty care
BSPHS: Barriers to Seeking Psychological Help Scale
IQR: interquartile ranges
DUP: duration of untreated psychosis
CHRp: clinical high risk of psychosis
FEP: first episode of psychosis
VSR: visual route timeline

## References

[1] Marshall M; Lewis S; Lockwood A; Drake R; Jones P; Croudace T Association between Duration of Untreated Psychosis and Outcome in Cohorts of First-Episode Patients. Arch. Gen. Psychiatry 2005, 62, 975. 16143729 10.1001/archpsyc.62.9.975

[2] Boonstra N; Klaassen R; Sytema S; Marshall M; De Haan L; Wunderink L; Wiersma D Duration of Untreated Psychosis and Negative Symptoms—A Systematic Review and Meta-Analysis of Individual Patient Data. Schizophr. Res 2012, 142, 12–19. 23025994 10.1016/j.schres.2012.08.017

[3] O’Keeffe D; Kinsella A; Waddington JL; Clarke M 20-Year Prospective, Sequential Follow-up Study of Heterogeneity in Associations of Duration of Untreated Psychosis with Symptoms, Functioning, and Quality of Life Following First-Episode Psychosis. Am. J. Psychiatry 2022, 179, 288–297. 35360921 10.1176/appi.ajp.2021.20111658

[4] Bhui K; Ullrich S; Coid JW Which Pathways to Psychiatric Care Lead to Earlier Treatment and a Shorter Duration of First-Episode Psychosis? BMC Psychiatry 2014, 14, 72. 24620939 10.1186/1471-244X-14-72 PMC3984674

[5] Martin I.d.S.; Ciccone Giacon BC; Giacchero Vedana KG; Guidorizzi Zanetti AC; Fendrich L; Frari Galera SA Where to Seek Help? Barriers to Beginning Treatment during the First-Episode Psychosis. Int. J. Nurs. Sci 2018, 5, 249–254. 31406833 10.1016/j.ijnss.2018.06.007 PMC6626253

[6] Kirkbride JB; Hameed Y; Ankireddypalli G; Ioannidis K; Crane CM; Nasir M; Kabacs N; Metastasio A; Jenkins O; Espandian A; The Epidemiology of First-Episode Psychosis in Early Intervention in Psychosis Services: Findings from the Social Epidemiology of Psychoses in East Anglia [SEPEA] Study. Am. J. Psychiatry 2017, 174, 143–153. 27771972 10.1176/appi.ajp.2016.16010103 PMC5939990

[7] Simon GE; Coleman KJ; Yarborough BJH; Operskalski B; Stewart C; Hunkeler EM; Lynch F; Carrell D; Beck A First Presentation with Psychotic Symptoms in a Population-Based Sample. Psychiatr. Serv 2017, 68, 456–461. 28045349 10.1176/appi.ps.201600257 PMC5811263

[8] Allan SM; Hodgekins J; Beazley P; Oduola S Pathways to Care in At-Risk Mental States: A Systematic Review. Early Interv. Psychiatry 2020, 15, 1092–1103. 33047505 10.1111/eip.13053

[9] Anderson KK; Fuhrer R; Malla AK The Pathways to Mental Health Care of First-Episode Psychosis Patients: A Systematic Review. Psychol. Med 2010, 40, 1585–1597. 20236571 10.1017/S0033291710000371

[10] Cabassa LJ; Piscitelli S; Haselden M; Lee RJ; Essock SM; Dixon LB Understanding Pathways to Care of Individuals Entering a Specialized Early Intervention Service for First-Episode Psychosis. Psychiatr. Serv 2018, 69, 648–656. 29493414 10.1176/appi.ps.201700018 PMC6007867

[11] Gronholm PC; Thornicroft G; Laurens KR; Evans-Lacko S Mental Health-Related Stigma and Pathways to Care for People at Risk of Psychotic Disorders or Experiencing First-Episode Psychosis: A Systematic Review. Psychol. Med 2017, 47, 1867–1879. 28196549 10.1017/S0033291717000344

[12] Marino L; Scodes J; Ngo H; Nossel I; Bello I; Wall M; Dixon L Determinants of Pathways to Care among Young Adults with Early Psychosis Entering a Coordinated Specialty Care Program. Early Interv. Psychiatry 2019, 14, 544–552. 31502409 10.1111/eip.12877

[13] Rogler LH; Cortes DE Help-Seeking Pathways: A Unifying Concept in Mental Health Care. Am. J. Psychiatry 1993, 150, 554–561. 8465869 10.1176/ajp.150.4.554

[14] Polillo A; Foussias G; Wang W; Voineskos AN; Veras J; Davis-Faroque N; Wong AHC; Kozloff N Care Pathways and Initial Engagement in Early Psychosis Intervention Services among Youths and Young Adults. JAMA Netw. Open 2023, 6, e2333526. 37703014 10.1001/jamanetworkopen.2023.33526 PMC10500372

[15] Oluwoye O; Weeks DL Ethnoracial Differences in Family Members’ Early Contact with Formal and Informal Resources on the Pathway to Care during the Early Stages of Psychosis. Community Ment. Health J 2023, 60, 244–250. 37418116 10.1007/s10597-023-01163-5 PMC10993660

[16] Anderson KK; Flora N; Ferrari M; Tuck A; Archie S; Kidd S; Tang T; Kirmayer LJ; McKenzie K Pathways to First-Episode Care for Psychosis in African-, Caribbean-, and European-Origin Groups in Ontario. Can. J. Psychiatry 2015, 60, 223–231. 26174526 10.1177/070674371506000504 PMC4484691

[17] van der Ven E; Jones N; Bareis N; Scodes J; Dambreville R; Ngo H; Mathai CM; Bello I; Martínez-Alés G; Mascayano F; An Intersectional Approach to Ethnoracial Disparities in Pathways to Care among Individuals with Psychosis in Coordinated Specialty Care. JAMA Psychiatry 2022, 79, 790. 35767311 10.1001/jamapsychiatry.2022.1640 PMC9244772

[18] Friedman BR; Durán DK; Nestsiarovich A; Tohen M; Lenroot RK; Bustillo JR; Crisanti AS Characteristics of Hispanics Referred to Coordinated Specialty Care for First-Episode Psychosis and Factors Associated with Enrollment. Psychiatr. Serv 2021, 72, 1407–1414. 34074143 10.1176/appi.ps.202000798

[19] Ferrari M; Flora N; Anderson KK; Haughton A; Tuck A; Archie S; Kidd S; McKenzie K Gender Differences in Pathways to Care for Early Psychosis. Early Interv. Psychiatry 2016, 12, 355–361. 27017924 10.1111/eip.12324

[20] Large MM; Nielssen O Gender Differences in the Duration of Untreated Psychosis. J. Nerv. Ment. Dis 2008, 196, 171–172. 18277228 10.1097/NMD.0b013e318162aa8b

[21] Gulliver A; Griffiths KM; Christensen H Perceived Barriers and Facilitators to Mental Health Help-Seeking in Young People: A Systematic Review. BMC Psychiatry 2010, 10, 113. 21192795 10.1186/1471-244X-10-113 PMC3022639

[22] Hasan AA; Musleh M Barriers to Seeking Early Psychiatric Treatment amongst First-Episode Psychosis Patients: A Qualitative Study. Issues Ment. Health Nurs 2017, 38, 669–677. 28485998 10.1080/01612840.2017.1317307

[23] Borgogna NC; Aita SL; Trask CL; Moncrief GG Psychotic Disorders in College Students: Demographic and Care Considerations. Psychosis 2023, 15, 229–239.

[24] Topkaya N; Şahin E; Meydan B The Development, Validity, and Reliability of the Barriers to Seeking Psychological Help Scale for College Students. Int. J. High. Educ 2016, 6, 48.

[25] Malla AK; Norman RMG; Manchanda R; Towsend L Symptoms, Cognition, Treatment Adherence and Functional Outcome in First-Episode Psychosis. Psychol. Med 2002, 32, 1109–1119. 12214790 10.1017/s0033291702006050

[26] Ferrari M; Pawliuk N; Pope M; MacDonald K; Boruff J; Shah J; Malla A; Iyer SN A Scoping Review of Measures Used in Early Intervention Services for Psychosis. Psychiatr. Serv 2022, 74, 523–533. 36321318 10.1176/appi.ps.202100506

[27] Archie S; Akhtar-Danesh N; Norman R; Malla A; Roy P; Zipursky RB Ethnic Diversity and Pathways to Care for a First Episode of Psychosis in Ontario. Schizophr. Bull 2008, 36, 688–701. 18987101 10.1093/schbul/sbn137 PMC2894595

[28] R Core Team. R: A Language and Environment for Statistical Computing*, Version 4.3.0*; R Core Team: Vienna, Austria, 2023.

[29] Anderson KK; Fuhrer R; Wynant W; Abrahamowicz M; Buckeridge DL; Malla A Patterns of Health Services Use prior to a First Diagnosis of Psychosis: The Importance of Primary Care. Soc. Psychiatry Psychiatr. Epidemiol 2013, 48, 1389–1398. 23429939 10.1007/s00127-013-0665-3

[30] Reynolds N; Wuyts P; Badger S; Fusar-Poli P; McGuire P; Valmaggia L The Impact of Delivering GP Training on the Clinical High Risk and First-Episode Psychosis on Referrals and Pathways to Care. Early Interv. Psychiatry 2014, 9, 459–466. 24602226 10.1111/eip.12126

[31] Sutton M; O’Keeffe D; Frawley T; Madigan K; Fanning F; Lawlor E; Roche E; Kelly A; Turner N; Horenstein A; Feasibility of a Psychosis Information Intervention to Improve Mental Health Literacy for Professional Groups in Contact with Young People. Early Interv. Psychiatry 2017, 12, 234–239. 28102617 10.1111/eip.12410

[32] Calderon V; Cain R; Torres E; Lopez SR Evaluating the Message of an Ongoing Communication Campaign to Reduce the Duration of Untreated Psychosis in a Latinx Community in the United States. Early Interv. Psychiatry 2021, 16, 147–152. 33768718 10.1111/eip.13140

[33] Ferrara M It’s time to think of women: Mental health services for first-episode psychosis. Psychiatr. Times 2022, 9, 17.

[34] Lai HJ; Lien YJ; Chen KR; Lin YK The Effectiveness of Mental Health Literacy Curriculum among Undergraduate Public Health Students. Int. J. Environ. Res. Public Health 2022, 19, 5269. 35564671 10.3390/ijerph19095269 PMC9104026

[35] Morgan C; Mallett R; Hutchinson G; Leff J Negative Pathways to Psychiatric Care and Ethnicity: The Bridge between Social Science and Psychiatry. Soc. Sci. Med 2004, 58, 739–752. 14672590 10.1016/s0277-9536(03)00233-8

